# Targeted Strategies to Modulate Stem-Cell-Relevant Pathways

**DOI:** 10.1155/2016/1859456

**Published:** 2016-09-15

**Authors:** Jonathan W. Lowery, James A. Ankrum, Shoichiro Kokabu, Renjing Liu

**Affiliations:** ^1^Division of Biomedical Science, Marian University College of Osteopathic Medicine, Indianapolis, IN, USA; ^2^Department of Biomedical Engineering, University of Iowa, Iowa City, IA, USA; ^3^Fraternal Order of Eagles Diabetes Research Center, Pappajohn Biomedical Institute, University of Iowa, Iowa City, IA, USA; ^4^Division of Molecular Signaling and Biochemistry, Department of Health Improvement, Kyushu Dental University, 2-6-1 Manazuru, Kokurakita-ku, Kitakyusyushi, Fukuoka 803-8580, Japan; ^5^Department of Oral and Maxillofacial Surgery, Faculty of Medicine, Saitama Medical University, 38 Morohongo, Moroyama-machi, Iruma-gun, Saitama 350-0495, Japan; ^6^Agnes Ginges Laboratory for Diseases of the Aorta, Centenary Institute, Camperdown, NSW, Australia; ^7^Sydney Medical School, University of Sydney, Sydney, NSW, Australia

Modulation of stem cell behavior is of significant interest to the biomedical community and could lead to novel therapeutic advances in treating disease. Achieving this goal requires specific strategies that manipulate the pathways regulating stem cell plasticity and behavior. The accumulating evidence indicates that just a few main signaling pathways regulate most types of stem cells, which suggests that strategies that modulate one type of stem cell might hold broad usefulness. However, as stem cell research becomes more and more specialized, investigators studying a particular pathway or behavior in one specialty can miss a breakthrough advancement made in another specialty.

In this special issue we have collected reports and reviews of pathways that are critical to regulating the function and fate of mesenchymal stem cells (MSCs), induced pluripotent stem cells (IPSCs), and endothelial progenitor cells (EPCs). While each report is focused on the fate and function of a particular type of progenitor cell or a particular pathway, mechanisms at play in one cell type may be directly relevant to other cell types as well.

The multipotent nature of MSCs makes them an attractive cellular source for regenerative medicine. While many reports exist describing the potential of MSC to repair damaged tissues following trauma, our understanding of the role of MSC in repair of polytrauma, that is, in tissues suffering more than two injuries, is still in its infancy. In this special issue, M. Huber-Lang and colleagues provided a summary of studies that shed light on the potential of MSC as a therapeutic target for treatment of polytrauma. Moreover, the authors present examples that add to both sides of the debate on whether MSC are “actors” that drive tissue regeneration or are “targets” for attacks by the immune system following polytrauma.

S. Kokabu et al. also focus on MSCs, examining the reciprocal relationship between differentiation of this cell type into osteogenic versus adipogenic lineages. Particular attention is paid to the function of the transcriptional regulator Transducin-Like Enhancer of Split 3 (TLE3), which has recently been implicated in regulating the commitment between these two lineages. Additionally, S. Kokabu and colleagues propose future areas of research which may lead to the ability to control adipogenic versus osteogenic differentiation in the bone marrow microenvironment.

Related to this, J. W. Lowery et al. survey the strategies that are available to modulate the Bone Morphogenetic Protein (BMP) signaling pathway, which potently induces both osteogenic and adipogenic differentiation of MSCs. The authors detail the currently available natural and engineered ligands, extracellular antagonists, ligand traps, and kinase inhibitors. Numerous examples of each strategy in specific settings and applications are presented. J. W. Lowery and colleagues also propose future areas for study in order to advance the ability to control behavior of MSCs, other stem cell populations, and somatic cells alike.

J. Zhao et al. examine the ability of late-outgrowth EPCs (LO-EPCs) to home to sites of injury after intravenous infusion via a series of* in vitro* experiments. LO-EPCs are capable of differentiating into endothelial cells, but are a rare cell type in circulation, making their* ex vivo* expansion necessary prior to therapy. In contrast to leukocytes and MSC which exhibit enhanced adhesion to inflamed endothelium, J. Zhao and colleagues reported no enhancement in LO-EPC adhesion in inflamed* in vitro* conditions. However, attachment was enhanced when the subcellular extracellular matrix was exposed. Disruption of endothelial barrier integrity by subconfluent seeding or incubation with anti-VE cadherin blocking antibodies resulted in increased LO-EPC adhesion, which the authors go on to show that it appears to be dominated by adhesion to fibronectin and vitronectin in the ECM. Thus, in contrast to MSC and leukocytes, disruption of endothelial integrity appears to be critical to facilitate LO-EPC homing.

Finally, P. Nagaria et al. examine how the method of conferring pluripotency affects the DNA damage response in cord blood myeloid progenitors and fibroblasts. The authors find that, in contrast to standard methods, a high-fidelity stromal-activated method results in IPSCs that closely resemble embryonic stem cells in their ability to repair double-stand DNA damage via non-homologous end joining and in their expression of c-MYC-mediated transcriptional signature. These findings are highly relevant to investigators working in the IPSC field and are potentially applicable to the safe clinical translation of IPSC-based therapies in patients.

*Jonathan W. Lowery* *Jonathan W. Lowery* *James A. Ankrum* *James A. Ankrum* *Shoichiro Kokabu* *Shoichiro Kokabu* *Renjing Liu* *Renjing Liu*

